# Enhancing Trauma Care: A Machine Learning Approach with XGBoost for Predicting Urgent Hemorrhage Interventions Using NTDB Data

**DOI:** 10.3390/bioengineering11080768

**Published:** 2024-07-30

**Authors:** Jin Zhang, Zhichao Jin, Bihan Tang, Xiangtong Huang, Zongyu Wang, Qi Chen, Jia He

**Affiliations:** 1 School of Health Sciences and Engineering, University of Shanghai for Science and Technology, Shanghai 200000, China; 233352431@st.usst.edu.cn (J.Z.); 232472769@st.usst.edu.cn (X.H.); zongyu79727@126.com (Z.W.); 2 Department of Health Statistics, Naval Medical University, Shanghai 200433, China; jzc109@hotmail.com; 3 Department of Health Management, Naval Medical University, Shanghai 200433, China; mangotangbihan@126.com

**Keywords:** trauma, hemorrhage, machine learning, XGBoost, urgent intervention, National Trauma Data Bank

## Abstract

Objective: Trauma is a leading cause of death worldwide, with many incidents resulting in hemorrhage before the patient reaches the hospital. Despite advances in trauma care, the majority of deaths occur within the first three hours of hospital admission, offering a very limited window for effective intervention. Unfortunately, a significant increase in mortality from hemorrhagic trauma is primarily due to delays in hemorrhage control. Therefore, we propose a machine learning model to predict the need for urgent hemorrhage intervention. Methods: This study developed and validated an XGBoost-based machine learning model using data from the National Trauma Data Bank (NTDB) from 2017 to 2019. It focuses on demographic and clinical data from the initial hours following trauma for model training and validation, aiming to predict whether trauma patients require urgent hemorrhage intervention. Results: The XGBoost model demonstrated superior performance across multiple datasets, achieving an AUROC of 0.872 on the training set, 0.869 on the internal validation set, and 0.875 on the external validation set. The model also showed high sensitivity (77.8% on the external validation set) and specificity (82.1% on the external validation set), with an accuracy exceeding 81% across all datasets, highlighting its high reliability for clinical applications. Conclusions: Our study shows that the XGBoost model effectively predicts urgent hemorrhage interventions using data from the National Trauma Data Bank (NTDB). It outperforms other machine learning algorithms in accuracy and robustness across various datasets. These results highlight machine learning’s potential to improve emergency responses and decision-making in trauma care.

## 1. Introduction

Trauma is one of the leading causes of death globally, accounting for 9.2% of all deaths worldwide and 10.9% of disability-adjusted life years [1]. The majority of these deaths are due to hemorrhage, with 33–56% occurring before hospital arrival [2]. Despite significant advancements in trauma management over the past two decades, uncontrolled bleeding remains the primary cause of preventable deaths among trauma patients, accounting for 40% of such deaths [3]. Most deaths due to hemorrhage occur within the first three hours before hospital admission, leaving a very limited window for effective intervention [4]. Unfortunately, the main reason for the significant increase in mortality from hemorrhagic trauma is the delay in bleeding control [5,6,7]. Therefore, early control of bleeding has become the primary goal in the treatment of patients with major hemorrhage.

Current trauma resuscitation approaches focus primarily on hemorrhage control, involving three key interventions: massive transfusion, endovascular embolization, and bleeding control surgery [8]. According to the Clinical Practice Guidelines (CPG) for major bleeding, balanced transfusion is recommended [9]. The current common trauma resuscitation strategy is the Massive Hemorrhage Protocol (MHP) [10]. In large trauma centers, resuscitation of hemorrhagic trauma patients with compromised physiology involves using plasma, platelets, and red blood cells (RBCs) in a 1:1:1 ratio [11]. Additionally, performing endovascular embolization on patients is crucial. Endovascular embolization, a common non-surgical treatment in interventional radiology [12], aims to deliberately occlude vessels to treat injured or diseased vascular systems [13]. Prior to the availability of endovascular embolization, treating hemorrhagic patients relied primarily on transfusions. However, in modern medical practice, endovascular embolization has become the preferred treatment method in some cases, as it allows for rapid and effective control of bleeding using minimally invasive techniques [14]. Reports indicate that the overall procedural success rate for gastrointestinal bleed embolization is between 93 and 100%, with clinical success rates ranging from 51 to 88% [15]. At the same time, it has been recognized that excessive fluid resuscitation can adversely affect hemostasis. To prevent the deadly triad of acidosis, coagulopathy, and hypothermia, bleeding control surgery is increasingly advocated [16]. For instance, Rotondo et al. in 1993 found that damage control laparotomy offers a significant survival advantage for the most severely injured patient subgroup compared to definitive laparotomy [17].

In recent years, breakthroughs in machine learning have provided more accurate diagnostic algorithms, offering personalized treatment for patients and helping physicians improve diagnostic accuracy [18]. Machine learning, a branch of computer science, involves the application of mathematical, statistical techniques, and computational algorithms. This field employs artificial intelligence algorithms to analyze variables in datasets and identify patterns to predict specific outcomes of interest [19]. To date, predicting the need for massive transfusion has been well established, but the application of machine learning in trauma-related emergency bleeding interventions (such as massive bleeding, endovascular embolization, or bleeding control surgery) is still relatively limited. Therefore, this article proposes a machine learning model that uses information from the first four hours before hospital admission to predict the need for emergency bleeding intervention.

## 2. Materials and Methods

### 2.1. Dataset

The National Trauma Data Bank (NTDB) of the United States collects millions of data records annually from nearly 700 hospitals, making it the largest trauma registry institution to date. The American College of Surgeons mandates that all level I and II trauma centers submit data to the NTDB, facilitating the formation of a more representative dataset. Currently, this dataset encompasses over 95% of all level I trauma centers in the United States [20].

This study utilizes the NTDB datasets starting from 2017, primarily due to an update in data format implemented that year. Specifically, the 2017 and 2018 NTDB datasets, which include 2,041,706 patients, were used for model development, hyperparameter tuning, and internal validation. The 2019 NTDB dataset, comprising 1,097,190 patients, was used for external validation of the model.

### 2.2. Dataset Preprocessing

**Figure 1:**
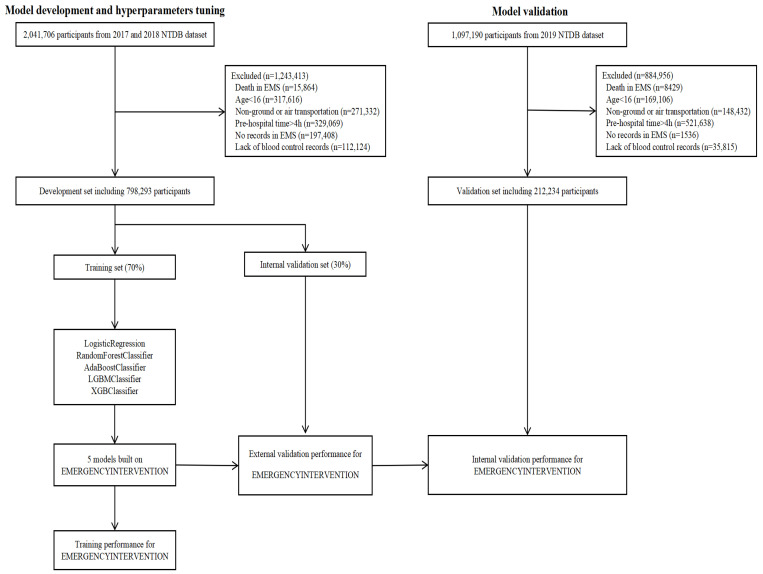
The flow chart of model development and validation.

All trauma patients aged 16 years or older, who were transported to any trauma center within four hours of injury via ground or air ambulance, were considered potentially eligible for the study. Patients who died in Emergency Medical Services (EMS), those without any EMS records, and those missing critical information on transfusions, vascular embolization, or bleeding control surgeries were excluded. Ultimately, 798,293 participants were included in the development set, and 212,234 participants were included in the validation set. The selection process of the patients is illustrated in Figure 1.

Emergency bleeding interventions are defined as meeting at least one of the following criteria: massive transfusion (MT), vascular embolization, or a type of bleeding control surgery. Massive transfusion is defined as the requirement of 1250 milliliters or 5 units of blood within 4 h [21]. Patients who did not undergo vascular embolization are considered not to need this intervention, while all others are deemed to require it. Similarly, patients who did not undergo bleeding control surgery are considered not to need this intervention, whereas those who underwent abdominal, thoracic, sternotomy, limb, neck, amputation, or other types of skin or soft tissue bleeding control surgeries are considered to require this intervention.

In the process of model development, the selection of variables is particularly crucial. If a model contains too many variables, it may perform well during training—known as overfitting—but it tends to perform poorly when dealing with new or unseen data. Conversely, if the model includes too few variables, it may fail to capture essential relationships within the data, potentially affecting performance on training data and leading to underfitting in test data. Seymour et al. (2021) emphasized the importance of vital signs and injury mechanisms in optimizing prehospital emergency responses and improving patient clinical management [22]. Similarly, Ayman et al. (2021) noted the impact of patients’ clinical characteristics and injury patterns on treatment outcomes [23]. Furthermore, Kang et al. (2022) developed an acute hemorrhage control model using a varying number of input features, gradually constructing three machine learning models of increasing complexity. Starting from a basic model that included only fundamental patient information and vital signs, they progressively incorporated parameters such as the Glasgow Coma Scale, significantly enhancing the model’s performance [24]. Given these prior studies and our research objectives, we need to meticulously select variables to be manually collected within a limited timeframe, controlling for the number and complexity of these variables, with all data being recordable within four hours before an emergency occurs. Therefore, we selected gender, age, seven vital signs, and eight injury patterns as candidate variables. The candidate variables are detailed in Table 1.

### 2.3. Model Development

To assess whether patients require emergency hemorrhage interventions, this study compared five machine learning models including logistic regression, random forest, Adaptive Boosting (AdaBoost), Gradient Boosting (LightGBM), and Extreme Gradient Boosting (XGBoost) [25,26,27,28]. In employing random forest, AdaBoost, LightGBM, and XGBoost, we utilized 100 trees with a maximum depth of five to balance model performance and computational efficiency. After a comprehensive evaluation of the performance of each model, XGBoost was chosen as the primary development model for predicting the need for emergency hemorrhage interventions.

XGBoost is an advanced ensemble learning method based on tree boosting techniques that incrementally refines the predictions by addressing the residuals or errors from earlier models and integrating these improvements into a finely tuned final prediction. This algorithm is highly regarded for its exceptional predictive performance, effectively managing both categorical and numerical inputs, significantly reducing the need for data preprocessing, and efficiently handling data imbalances while maintaining computational efficiency [29]. Notably, XGBoost can process data with missing values and offers branching capabilities, making it highly effective even when comprehensive initial data are lacking [30,31]. Its excellence in continuous integration improvement and ensuring optimized internal operations has made XGBoost a preferred tool among global data science and machine learning experts.

The patient data used were sourced from the 2017 and 2018 datasets of the National Trauma Data Bank (NTDB), which were used to construct the development set. Patients were randomly assigned to a training set (70%) and an internal validation set (30%) for model development. Ten-fold cross-validation was implemented on the training set, and the model’s hyperparameters were optimized using grid search. The adjusted hyperparameters included a learning rate of 0.3593813663804626, a minimum loss reduction required for further partitioning of the tree leaves set at 0.04, a maximum depth of the trees at 5, regularization parameters reg_lambda at 50 and reg_alpha at 0.5, maximum iteration number at 100, and a subsample ratio of training instances at 0.92, with other hyperparameters remaining at default settings. The reproducibility, portability, and generalizability of the model were evaluated on the training set and validated both internally and externally.

Furthermore, to gain a deeper understanding of the performance of the risk prediction model, this study incorporated SHAP (Shapley Additive Explanations), a game-theoretic approach first introduced in the 1950s, to analyze the impact of predictive factors based on Shapley values. Shapley values can be positive or negative; a positive value indicates an increased likelihood of needing emergency hemorrhage intervention, while a negative value suggests a decreased likelihood. We conducted a detailed analysis of the roles of various predictive factors through visualizations at both the global (entire dataset) and local (individual patient) levels [32].

### 2.4. Statistical Analysis Methods

In this study, continuous data are presented as means with their standard deviations (SD), while categorical data are shown as frequencies and percentages (%). For continuous data, if the data are normally distributed and have equal variances, the *t*-test is used to analyze the differences; otherwise, the Wilcoxon rank-sum test is applied. Differences in categorical data are assessed using the Pearson chi-square test. The discriminative ability of the models is evaluated by calculating the area under the Receiver Operating Characteristic (ROC) curve and other performance metrics, including sensitivity, specificity, accuracy, positive predictive value (PPV), and negative predictive value (NPV). Additionally, calibration plots were generated to compare the predictive probabilities of the five models with the actual probabilities, and Decision Curve Analysis (DCA) was employed to evaluate the clinical utility of these models.

## 3. Results

### 3.1. Patient Characteristics

Table 2 presents the baseline characteristics of patients in the training set, internal validation set, and external validation set. It is noteworthy that while most variables show statistically significant differences across these three large sample groups, the differences are relatively minor.

### 3.2. Model Performance

Table 3 extensively presents the comparative results of five models across multiple performance metrics. Particularly, the AUROC, a key indicator for assessing a model’s capability to differentiate between positive and negative classes, shows significantly high values indicating superior classification performance. Notably, XGBoost demonstrates excellent AUROC values across the training set, internal validation set, and external validation set, fully showcasing its outstanding predictive capabilities. Furthermore, specificity and sensitivity, important criteria for evaluating a model’s ability to distinguish between negative and positive classes, often show that XGBoost performs better than other models, occasionally even achieving the best results. Additionally, PPV and NPV, which measure the accuracy of predicting positive and negative classes, respectively, show that while XGBoost may not always offer the ideal PPV in some datasets, its overall performance, including NPV, is generally well balanced. Accuracy, an intuitive indicator of overall predictive accuracy, is consistently high for XGBoost across all datasets, further demonstrating its robust generalization capabilities. In summary, these comprehensive evaluation results highlight the status of XGBoost as a reliable preferred model across various datasets.

**Figure 2:**
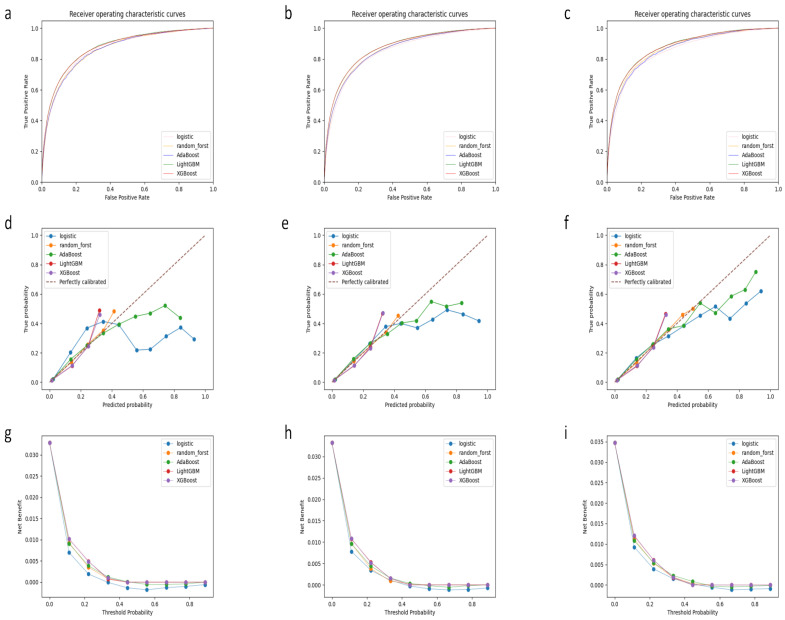
ROC curves, calibration curves, and decision curves of the 5 models. (**a**) ROC curves in the training set. (**b**) ROC curves in the internal validation set. (**c**) ROC curves in the external validation set. (**d**) Calibration curves in the training set. (**e**) Calibration curves in the internal validation set. (**f**) Calibration curves in the external validation set. (**g**) Decision curves in the training set. (**h**) Decision curves in the internal validation set. (**i**) Decision curves in the external validation set.

Figure 2 presents the ROC curves, calibration curves, and decision curves for five models across training, internal validation, and external validation sets. It is evident that the ROC curves of the XGBoost model (training set: 0.872, internal validation set: 0.869, external validation set: 0.875) are predominantly higher than those of other models, demonstrating its significant classification performance. Calibration curves, which assess the consistency between predicted probabilities and actual occurrence probabilities, show a slight advantage in the Random Forest model; however, the calibration curve of the XGBoost model also exhibits good predictive consistency, closely aligning with the y = x diagonal. Moreover, decision curve analysis reveals the net benefits of predicting positive outcomes across various threshold probabilities. The XGBoost model offers a broader range of net benefits at different threshold probabilities, such as 0.1 to 0.9, supporting its application advantages in clinical decision-making, where the potential benefits of reducing misdiagnoses significantly outweigh the costs of incorrect diagnoses.

### 3.3. Feature Importance

**Figure 3:**
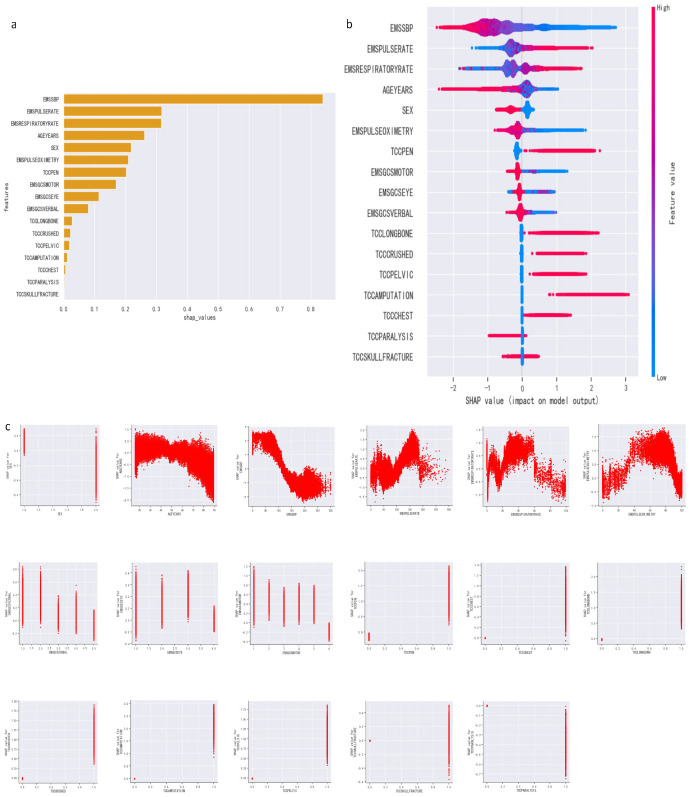
In the training dataset, we employed the SHAP method to provide a global explanation of the model predicting hemorrhagic emergency interventions. (**a**) Displays a bar chart summarizing the average SHAP values for each variable. (**b**) Presents the SHAP summary dot plot, where each dot represents an individual patient, positioned according to the SHAP value of the variable and colored to reflect the actual value of the feature; the dots are vertically stacked to indicate the density of data. (**c**) Shows the SHAP dependence plots, where each plot reveals the relationship between the actual values of the variables and their SHAP values, with each dot also representing a patient.

The SHAP (Shapley Additive Explanations) summary plot in Figure 3a vividly displays the contributions of various variables to the XGBoost model’s prediction of the need for urgent hemorrhage interventions. By calculating the average absolute values of contributions from diverse variables, we identified blood pressure, pulse, respiratory rate, age, and gender as the five key factors. Figure 3b illustrates how the SHAP values of these variables influence the necessity for emergency bleeding interventions. Moreover, Figure 3c reveals the relationship between the SHAP values of these variables and their actual measurements. Notably, we observed that higher blood pressure typically indicates a lower need for emergency hemorrhage interventions, reflecting the negative impact of blood pressure on outcomes. Concurrently, gender, age, seven vital signs, and two injury patterns also appear to negatively affect the prediction results. Conversely, an additional six injury patterns positively contribute to the predictive outcomes. These visualizations collectively provide a clear and quantifiable insight into how different clinical and demographic factors affect the model’s predictions regarding the need for emergency interventions in trauma patients.

## 4. Discussion

In this study, we utilized data from the National Trauma Data Bank (NTDB) to develop an XGBoost-based machine learning model for predicting whether trauma patients require emergency hemorrhage intervention. Particular attention was given to the critical first four hours after trauma, a key period for improving patient survival rates. By conducting a thorough analysis and optimization of data from 2017 to 2018 and validating with 2019 data, we demonstrated the effectiveness of machine learning in clinical prediction. The XGBoost model performed exceptionally well across all test datasets, particularly in terms of AUROC values, showcasing its superior performance in classification tasks. Additionally, the model maintained a good balance in specificity, sensitivity, and predictive values, consistently outperforming other comparative models. Decision Curve Analysis (DCA) also revealed that the XGBoost model provided significant net benefits across multiple probability thresholds, further highlighting its practical value in supporting clinical decisions. In the emergency management of traumatic hemorrhage, accurate identification of influencing factors is crucial for the formulation of treatment plans. While machine learning techniques demonstrate remarkable accuracy in prediction and classification, their internal mechanisms often remain elusive, resembling a “black box” that is difficult to intuitively comprehend. To address this challenge, we have adopted the SHAP (SHapley Additive exPlanations) method, which not only preserves the robust learning capabilities of machine learning models but also effectively addresses the issue of interpretability of model outcomes. By utilizing SHAP values, we have analyzed and identified key factors that contribute to the accuracy of predictions, such as blood pressure, pulse rate, and respiratory rate. This is crucial for making critical decisions in emergency situations. Compared to other models such as logistic regression, random forest, AdaBoost, and LightGBM, XGBoost exhibited higher AUROC values on different datasets, with values of 0.872, 0.869, and 0.875, respectively. This effective ability for identification and decision-making is particularly significant in handling traumatic cases [33,34,35].

This study primarily explores the predictive capabilities of machine learning models in trauma care, particularly regarding the prediction of urgent hemorrhage interventions at critical moments. This research direction echoes the findings of Hawkins et al. (2023), who emphasized the importance of timely interventions in trauma care but did not explore the potential of machine learning to predict these intervention needs [36]. Unlike Hawkins et al., we utilized the XGBoost model to predict specific intervention requirements and demonstrated its outstanding specificity and sensitivity across multiple validation phases. Additionally, Wang et al. (2021) share a similar perspective with us, also utilizing various models to predict bleeding in trauma patients [37]. Among them, the XGBoost model was more effective in handling data imbalance and providing more accurate predictions. In Wang’s research report, the XGBoost model demonstrated high predictive performance with an AUROC value reaching approximately 0.81. Additionally, Feng et al. (2023) highlighted the challenges of applying machine learning models in real clinical settings, particularly in accurately predicting the transfusion needs of trauma patients. They defined two time thresholds—4 h and 24 h—for clinical observation, using the XGBoost model for predictions. Their study found that the model performed similarly at both the 4-h and 24-h transfusion thresholds, with an AUROC value of 0.76, indicating good predictive performance [21]. When we applied the XGBoost model for similar research, we also achieved remarkable results, with our model achieving an AUROC value of approximately 0.87. This finding not only indicates the high reliability of the XGBoost model in handling such predictive tasks, but also further validates our work in model tuning and feature selection. Concurrently, Liu et al. (2023) also employed machine learning models to predict surgical needs in trauma cases, focusing mainly on general surgical requirements without delving into specific types of emergency interventions [38]. Our research addresses this gap by not only predicting surgical needs but also categorizing the urgency and type of surgical and non-surgical interventions, providing a more detailed tool for clinical decision-making.

The exceptional performance of XGBoost is largely attributed to its effective methods for handling the common issue of imbalanced data in medical datasets [39]. The design of this model allows for continuous optimization based on training data, while meticulous data preprocessing and the selection of the most critical features effectively reduce data noise, thereby enhancing predictive accuracy [40]. These characteristics make XGBoost a powerful tool for supporting rapid and accurate decision-making in clinical settings, especially in trauma centers, where its application in emergency situations is crucial and could potentially save many lives [41]. Overall, this study not only demonstrates the effectiveness of XGBoost in determining whether trauma patients require urgent hemorrhage intervention but also sets new standards for the application of machine learning in trauma care, providing valuable insights for future clinical practice and research.

Despite the achievements of this study, there are several limitations. Firstly, the performance of the model heavily depends on the quality and completeness of the NTDB data. For instance, although we conducted meticulous work in feature selection, it is undeniable that these choices were constrained by the available data in the NTDB. Consequently, other important predictors for hemorrhage intervention may not have been included in the model. Additionally, any inaccuracies or missing information in the dataset could negatively impact the model’s predictive performance and generalizability. Furthermore, NTDB data primarily originate from Level I and II trauma centers in the United States, which may limit the model’s applicability in other regions, especially those with different healthcare infrastructures and patient demographics. Lastly, the model was developed and validated using data from 2017 to 2019. Since then, advances in trauma care practices and technologies may affect the current applicability of the model. Therefore, to maintain the model’s relevance, it is essential to continuously update and retrain it using the most recent data.

## 5. Conclusions

This study demonstrates the significant potential of the XGBoost model in predicting urgent hemorrhage interventions for trauma patients using the extensive data from the National Trauma Data Bank (NTDB). The results show that the XGBoost model surpasses other machine learning models in predictive accuracy and achieves higher AUROC values across multiple validation sets, particularly exhibiting notable advantages in emergency and trauma care scenarios. Through in-depth data analysis and strategic application of SHAP values, the model effectively highlights the importance of key predictors such as blood pressure, pulse rate, and respiratory rate, which are crucial for rapidly determining the need for significant interventions such as massive transfusions or emergency surgeries. Additionally, the study ensures the high accuracy and real-time clinical applicability of the model through precise data preprocessing and feature selection, meeting the ongoing demands for continual improvement and refinement of machine learning techniques in medical practice. Overall, the performance of the XGBoost model sets a new standard for the application of machine learning in trauma care and paves the way for future applications in other emergency care areas, expanding the role of machine learning in medical diagnostics and intervention planning. We encourage continued research and adoption of these technologies to advance clinical diagnostics and emergency medical responses.

## Figures and Tables

**Table 1:** The candidate variables for model development.

Candidate Variables	Variable in NTDB *
Gender	SEX
Age	AGEYEARS
Initial EMS Systolic Blood Pressure	EMSSBP
Initial EMS Pulse Rate	EMSPULSERATE
Initial EMS Respiratory Rate	EMSRESPIRATORYRATE
EMS Oxygen Saturation	EMSPULSEOXIMETRY
EMS GCS—Eye	EMSGCSEYE
EMS GCS—Verbal	EMSGCSVERBAL
EMS GCS—Motor	EMSGCSMOTOR
Trauma Center Critera: All penetrating injuries to head, neck, torso, and extremities proximal to elbow or knee	TCCPEN
Trauma Center Critera: Chest wall instability or deformity (e.g., flail chest)	TCCCHEST
Trauma Center Critera: Two or more proximal long-bone fractures	TCCLONGBONE
Trauma Center Critera: Crushed, degloved, mangled, or pulseless extremity	TCCCRUSHED
Trauma Center Critera: Amputation proximal to wrist or ankle	TCCAMPUTATION
Trauma Center Critera: Pelvic fracture	TCCPELVIC
Trauma Center Critera: Open or depressed skull fracture	TCCSKULLFRACTURE
Trauma Center Critera: Paralysis	TCCPARALYSIS

* As an abbreviation in the article.

**Table 2:** Baseline characteristics of the patients from the training set, internal validation set, and external validation set.

Characteristics	Training Set (*n* = 558,805)	Internal Validation Set (*n* = 239,488)	External Validation Set (*n* = 212,234)	*p* Value
Sex				0.007
Male	341,005 (61.02)	145,932 (60.93)	130,158 (61.33)	
Female	217,743 (38.97)	93,525 (39.05)	82,038 (38.65)	
Transport mode				<0.001
Ground	516,501 (92.43)	221,333 (92.42)	190,546 (89.78)	
Helicopter	41,677 (7.46)	17,887 (7.47)	21,161 (9.97)	
Fixed-wing	627 (0.11)	268 (0.11)	527 (0.25)	
TCCPEN, yes	23,213 (4.15)	9952 (4.16)	7334 (3.46)	<0.001
TCCCHEST, yes	1378 (0.25)	565 (0.24)	651 (0.31)	<0.001
TCCLONGBONE, yes	4106 (0.73)	1732 (0.72)	1707 (0.80)	0.002
TCCCRUSHED, yes	2947 (0.53)	1187 (0.50)	1469 (0.69)	<0.001
TCCAMPUTATION, yes	573 (0.10)	262 (0.11)	228 (0.11)	0.88
TCCPELVIC, yes	3088 (0.55)	1306 (0.55)	1368 (0.64)	<0.001
TCCSKULLFRACTURE, yes	1751 (0.31)	793 (0.33)	982 (0.46)	<0.001
TCCPARALYSIS, yes	2060 (0.37)	904 (0.38)	806 (0.38)	0.92
EMERGENCYINTERVENTION				<0.001
MT, yes	9265 (1.66)	3945 (1.65)	3658 (1.72)	
ANGIOEMBOLIZATION, yes	4838 (0.87)	1979 (0.83)	1876 (0.88)	
HMRRHGCTRLSURG, yes	13,904 (2.49)	5875 (2.45)	5388 (2.54)	
Age	52.47 (21.79)	52.43 (21.80)	52.17 (21.73)	<0.001
EMSSBP, mmHg	139.25 (29.38)	139.29 (29.50)	139.88 (29.85)	<0.001
EMSPULSERATE, n/minute	90.65 (20.92)	90.59 (20.85)	90.68 (21.22)	0.15
EMSRESPIRATORYRATE, n/minute	18.36 (4.94)	18.37 (4.96)	18.58 (5.11)	<0.001
EMSPULSEOXIMETRY, %	96.11 (6.68)	96.08 (6.80)	95.99 (6.48)	<0.001
EMSGCSEYE	3.78 (0.70)	3.78 (0.70)	3.78 (0.74)	<0.001
EMSGCSVERBAL	4.56 (1.01)	4.56 (1.01)	4.59 (1.04)	<0.001
EMSGCSMOTOR	5.69 (1.04)	5.69 (1.05)	5.69 (1.10)	<0.001
EMSTOTALGCS	14.04 (2.60)	14.04 (2.60)	14.05 (2.76)	<0.001

The continuous data were described by mean (SD), and categorical data were described by *n* (%).

**Table 3:** Performance metrics of the 5 models for predicting emergent bleeding intervention.

	AUROC	Spec	Sens	PPV	NPV	Accuracy
**Training set**						
Logistic regression	0.858	0.821	0.751	0.125	0.990	0.819
Random forest	0.857	0.796	0.762	0.113	0.990	0.795
Ada boost	0.858	0.800	0.763	0.115	0.990	0.799
XGB	0.872	0.826	0.764	0.130	0.990	0.824
LGB	0.873	0.816	0.775	0.125	0.991	0.815
**Internal validation set**						
Logistic regression	0.845	0.821	0.727	0.123	0.989	0.818
Random forest	0.856	0.794	0.766	0.113	0.990	0.793
Ada boost	0.855	0.803	0.752	0.116	0.990	0.801
XGB	0.869	0.823	0.763	0.129	0.990	0.821
LGB	0.870	0.817	0.771	0.127	0.990	0.815
**External validation set**						
Logistic regression	0.850	0.831	0.733	0.135	0.989	0.827
Random forest	0.865	0.801	0.778	0.123	0.990	0.800
Ada boost	0.861	0.806	0.763	0.124	0.990	0.804
XGB	0.875	0.821	0.778	0.135	0.990	0.819
LGB	0.877	0.815	0.786	0.133	0.991	0.814

XGB = extreme gradient boosting, LGB = light gradient boosting machine, AUROC = area under the receiver-operating characteristic, Spec = specificity, Sens = sensitivity, PPV = positive predictive value, NPV = negative predictive value.

## Data Availability

The NTDB datasets analyzed during our study are available in ACS Trauma Quality Programs, [https://www.facs.org/quality-programs/trauma/quality/national-trauma-data-bank/datasets/] (accessed on 24 June 2022). The final data used during the current study available from the corresponding author on reasonable request.
